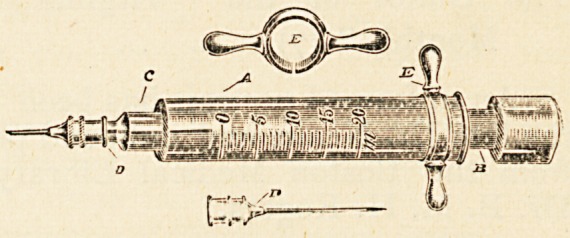# Notes on New Drugs and Preparations for the Sick

**Published:** 1902-12

**Authors:** 


### Notes on New Drugs and Preparations for the Sick.

We have received the following preparations from JAMES WOOLLEY, SONS & Co. Ltd., Manchester:

**Akolethe; Sol. Sodæ Phenatis; Decalcified Bone; Elixir Terpini Co.; Elixir Triferrin; Sanitary Rose Powder; Phenate of Soda Tooth-paste.**

The **Akolethe** is a solution of the sedative principles of opium of definite composition and uniform strength, equal to that of the Tinct. Opii. P.B. It is free from nauseous taste, and rarely produces any unpleasant after-effects. It has given excellent results, and is its own commendation—needing no other.

The **Solutio Sodæ Phenatis** is a strong antiseptic, resembling carbolic acid, but less caustic. When diluted in various degrees, it forms a useful application for all kinds of injuries to the skin and mucous membranes.

The **Phenate of Soda Tooth-paste** is a fragrant antiseptic for cleansing and preserving the teeth and gums.

The **Sanitary Rose Powder** has long been in use as a high-class toilet powder, with no irritating or pernicious properties. It is free from starch, is quite soluble, and acts as a deodorising antiseptic.

**Cancellous Tissue (Decalcified Bone).**—This substance seems well prepared, and, in one case in which we tried it, caused no inflammatory disturbance. Materials used for filling cavities made in operating upon bone, etc., have not met with much success, but Cancellous Tissue would certainly seem worth a trial.

The **Elixir Terpini Co.** is a combination of two very useful drugs—terpin and heroin. Each fluid drachm contains one grain of terpin hydrate, and one forty-eighth of a grain of heroin hydrochloride. It is an elegant syrupy preparation, which should have a wide range of utility in bronchitic and other respiratory affections. The dose is given as one to three drachms, but as much as four drachms would contain only an ordinary dose of the heroin. These cough sedatives may of course be abused, but, on the other hand, they give a great degree of comfort such as quite justifies their frequent use.

The **Elixir Triferrin** is a palatable preparation of a paranucleinate of iron. The Triferrin contains 22 per cent. of iron in organic combination, with $2\frac{1}{2}$ per cent. of phosphorus. For adults the dose is four drachms. We cannot well have too many preparations of the indispensable drug ferrum: when one kind disagrees, it is convenient to resort to another with a different name; and the organic combinations sometimes give success where the inorganic iron salts have failed.

---

**Milk Chocolate.**—Cadbury Brothers Limited, Bournville, Birmingham.—This Chocolate is a confection of exquisite flavour, and a nourishing food of considerable value. It is made from cocoa, sugar, and milk from the pastures of Worcestershire. Analysis shows that it contains 30 per cent. of fats, 52 per cent. of sugar, and about 10 per cent. of albuminoids, with a very small proportion of indigestible fibre. It is entirely free from colouring matters and preservatives. It possesses excellent keeping qualities, and undergoes no deterioration in any climate. It is one of the best varieties of chocolate we have met with, and is in itself a fairly perfect food, combining the three essentials—fats, sugars, and albuminoids in proportion, to supply materials for a great amount of muscular and other work. No cyclist should be without it.

---

**Dolle's Aromatic Iron Milk.** (Lac. Ferri Aromat. Dolle) — The British Iron Milk Syndicate Limited, Savoy House, London, W.C.—This is an entirely new preparation of iron, which contains pyrophosphate of iron, suspended in water, with glycerine, spirit, and aromatics. It is a white fluid, resembling milk, but with little taste, except aromatics. It does not blacken the teeth; and is not likely to cause either dyspepsia or constipation. The dose is a teaspoonful to a tablespoonful, three times a day, after food. It is especially recommended in chlorotic cases, in dysmenorrhœa, neurasthenia, and debility, but above all, for rickety and scrofulous children. It is a kind of preparation which is likely to be very useful, especially for those patients who say they cannot take iron preparations in the usual forms.

---

**Palatinoids.**—OPPENHEIMER, SON & Co., London.—Pepsin, Pancreatin, Calcii Lactophosph. aa gr. j. (gastrodynic). One to three of these palatinoids, taken after meals, should give relief in most cases of functional dyspepsia not dependent on organic defect. The drugs are of good quality, and may be relied upon to retain their activity, as they are well preserved in this palatinoid form.

---

**Antiphlogistine.**—THE DENVER CHEMICAL MANUFACTURING Co., New York; 18 Jeffreys Road, Clapham Road, London, S.W.—This paste is composed of glycerine, boric acid, salicylic acid, iron carbonate, peppermint, gaultheria, eucalyptus, and iodine, combined with the base, silicate of alumina and magnesia.

**Thermofuge.**—Messrs. PARKE DAVIS & Co., London.—Under this name, a preparation similar to the above has been introduced: it is a stiff paste, composed of aluminium silicate, glycerine, boric acid, menthol, thymol, oil of eucalyptus, and ammonium iodide—an antiseptic combination for external use.

These external applications are designed to take the place of poultices, and to be used whenever poultices have been employed in cases of pneumonic and other internal congestions. They are first warmed, then spread on the skin in a fairly thick layer, and covered with cloth. They are easily applied and as easily removed, and have antiseptic and soothing effects. They are said to have all the virtues of the poultice without its vices, and to be well worthy of trial. The poultice has had its day: the new preparation takes its place.

---

**Petanelle.**—PATÉ, BURKE & Co., 6 Wool Exchange, London, E.C.—Petanelle Wool; Petanelle Nap; Petanelle Pads. These substances appear to be inferior to most surgical dressings in their power of absorbing discharges. When placed on water they do not sink, as most absorbent materials do; and, applied to a wound, we found that a large quantity of the discharge escaped under the dressing, instead of being absorbed thereby. The dark brown colour of the material will probably also militate against their use for surgical dressings, though this is a minor point, compared with their lack of absorption. The Petanelle Nap is elastic, and would make a good padding for splints.

**Phillips' Milk of Magnesia.**—THE CHARLES H. PHILLIPS CHEMICAL CO., New York; 14 Henrietta Street, London, W.C. —This milky-looking fluid is designed for the treatment of oral acidity, which is well known to be one of the most potent factors in the production of caries, erosions, and sensitive dentine. It is odorless, practically tasteless, milk-like in appearance, and contains 24 grains of magnesium hydroxide ($M_gH_2O_2$) to the fluid ounce of distilled water. It is free from grit, homogeneous, and non-irritating; it will neutralise twice its volume of lemon juice, and it neutralises acid saliva so completely that the alkalinity is maintained for hours. It is advised that a teaspoonful be taken into the mouth two or three times daily, particularly just before retiring. It should be worked with tooth-brush, tongue, and lips into all the spaces about the teeth, which will then remain coated with an alkaline film, protecting them from the action of acids for several hours.

---

**All-glass Aseptic Hypodermic Syringe.**—BURROUGHS, WELLCOME & CO., London —DIRECTIONS: Draw into the syringe the required quantity of sterile water, remove the nozzle, drop in the "Tabloid" Hypodermic Product, *carefully* replace the nozzle, and assist solution by shaking.

The syringe should be sterilised before and after use. To prevent sticking, each part should be thoroughly dried before it is replaced.

Owing to the precision with which the various parts fit, no lubricant is necessary. For the same reason, the vacuum within the cylinder should not be tested by placing the finger over the nozzle and drawing back the piston. The recoil will fracture the knob.

The barrel, piston, and nozzle consist entirely of glass, the solid piston **B** gliding evenly within the barrel **A**. The nozzle **C** fits into the barrel **A** by means of a perfect plug joint, and takes a needle **D** of the usual pattern. The principle of this syringe is that of what is called by engineers "the plunger," instead of the ordinary piston, and it works exceedingly well. It is always ready for action, and cannot get out of order. It is quite a pleasure to have one of these all-glass syringes at our disposal. They are made in two sizes, for 15 and 20 minims respectively.

We have also recently seen a much larger syringe, with an all-glass plunger, for the injection of antitoxin.

---

**Vin Urané Pesqui.** Fassett & Johnson, 31 & 32 Snow Hill, London.—This uranated wine is prepared by Mr. Pesqui, of

Bordeaux, and is considered to be of much value in the treatment of diabetes. It is prepared by the addition of azotate of uranium, bromide of lithium, pepsine, quinine, and other drugs to an old Bordeaux wine (Medoc). It has a distinctly medicinal flavour, but is not unpleasant, and when diluted is an excellent thirst quencher. It is said that about ten bottles are usually necessary before the treatment can be considered complete, and that ten thousand deaths occur annually from diabetes in France, whereas they could have been prevented by means of the Vin Urané Pesqui! We can scarcely accept this statement as established fact.

## Figures and Tables

**Figure f1:**